# Creativity at rest: Exploring functional network connectivity of creative experts

**DOI:** 10.1162/netn_a_00317

**Published:** 2023-10-01

**Authors:** William Orwig, Roni Setton, Ibai Diez, Elisenda Bueichekú, Meghan L. Meyer, Diana I. Tamir, Jorge Sepulcre, Daniel L. Schacter

**Affiliations:** Department of Psychology, Harvard University, Cambridge, MA, USA; Gordon Center for Medical Imaging, Department of Radiology, Massachusetts General Hospital and Harvard Medical School, Boston, MA, USA; Department of Psychological and Brain Sciences, Dartmouth College, Hanover, NH, USA; Department of Psychology, Princeton University, Princeton, NJ, USA

**Keywords:** Creativity, fMRI, Functional connectivity, Distal simulation, Vividness

## Abstract

The neuroscience of creativity seeks to disentangle the complex brain processes that underpin the generation of novel ideas. Neuroimaging studies of functional connectivity, particularly functional magnetic resonance imaging (fMRI), have revealed individual differences in brain network organization associated with creative ability; however, much of the extant research is limited to laboratory-based divergent thinking measures. To overcome these limitations, we compare functional brain connectivity in a cohort of creative experts (*n* = 27) and controls (*n* = 26) and examine links with creative behavior. First, we replicate prior findings showing reduced connectivity in visual cortex related to higher creative performance. Second, we examine whether this result is driven by integrated or segregated connectivity. Third, we examine associations between functional connectivity and vivid distal simulation separately in creative experts and controls. In accordance with past work, our results show reduced connectivity to the primary visual cortex in creative experts at rest. Additionally, we observe a negative association between distal simulation vividness and connectivity to the lateral visual cortex in creative experts. Taken together, these results highlight connectivity profiles of highly creative people and suggest that creative thinking may be related to, though not fully redundant with, the ability to vividly imagine the future.

## Author Summary

This study sought to uncover the neurocognitive basis of creativity by comparing brain connectivity in a cohort of creative experts and controls. Using graph theory methods, we replicate past work showing reduced connectivity to the primary visual cortex in highly creative people. Additionally, we observed a negative association between vividness of distal future simulation and connectivity to the lateral visual cortex in creative experts. Taken together, these findings highlight connectivity profiles of highly creative people and suggest that creative thinking may be related to the ability to vividly imagine events in the distant future.

## INTRODUCTION

The neuroscience of creativity seeks to disentangle the complex brain processes that underpin the generation of novel ideas. Neuroimaging studies of functional connectivity, particularly functional magnetic resonance imaging (fMRI), have revealed individual differences in brain network organization associated with creative ability (Beaty et al., 2014; Takeuchi et al., 2012); however, much of the extant research is limited to laboratory measures of creative performance. Divergent thinking (DT) has been a central concept in creativity research since its first usage by Guilford (1950). The most common assessment of DT is the alternative uses task (AUT), involving the generation of novel uses for common objects. Behavioral studies have shown that DT, as assessed by the AUT, predicts both the quantity of self-reported creative achievements (Jauk et al., 2014) and the quality of expert-rated creative performance (Beaty et al., 2013), as well as the academic and creative successes of school-aged children (Plucker, 1999; Torrance, 1988). The predictive power of DT has fueled decades of empirical research on the neurocognitive basis of creativity (Cogdell-Brooke et al., 2020; Kim, 2008; Runco & Acar, 2012; Wu et al., 2015). Computerized methods for scoring AUT have led to widely accessible tools for assessing individual differences in creativity (Beaty & Johnson, 2021). While DT certainly captures some aspects of creativity, it is not all-encompassing and its psychometric merit has been the subject of some debate in the field (Forthmann et al., 2021; Zeng et al., 2011). To overcome these limitations, we focus our analyses on a cohort of notable creative experts.

Current theories suggest that creativity is not attributable to a single brain region; rather, novel ideas result from interactions between large-scale brain networks (Beaty et al., 2019). Among the most well studied of these functional brain networks, the default network (DN)—including midline and posterior inferior parietal regions—shows increased activation in the absence of an external stimulus (Raichle et al., 2001). DN activity has been linked with self-generated thought, such as mind wandering and imagination (Andrews-Hanna et al., 2014; Buckner et al., 2008). Both task-based and resting-state fMRI studies have contributed to current understanding of the neurocognitive basis of creativity. Task-based approaches have helped to uncover the relationship among networks during creative task performance (Beaty et al., 2018; Shi et al., 2018). It has been proposed that DN contributes to the generation of candidate ideas, while executive control networks exert top-down monitoring to meet specific task goals or constraints (Beaty et al., 2014, 2016). Resting-state functional connectivity is thought to reflect experience-dependent patterns of connectivity that relate to individual and group differences in behavior (Stevens & Spreng, 2014). Research on resting-state network organization in relation to creative cognition has investigated interindividual differences, finding that increased “hubness” (the presence of hubs that form connections between different communities) of DN regions was positively associated with creativity (Kenett et al., 2020). Additionally, a recent study from our group has identified a pattern of reduced resting-state connectivity to the visual cortex associated with increased DT (Orwig et al., 2021). One interpretation of these results is that highly creative people are more prone to engage in internally directed cognition in the absence of an external task. A primary aim of the present study was to replicate this finding of reduced connectivity to visual cortex in a cohort of creative experts.

While most participants in creativity research are drawn from the general population, some recent efforts involve the collection of neuroimaging data from creative professionals. The Big-C Project is a large-scale study seeking to identify behavioral and functional brain characteristics that distinguish exceptionally creative people. For instance, Anderson et al. (2022) found that highly creative individuals had higher local clustering coefficients during resting state and reduced local clustering while performing the AUT compared with less creative individuals. Furthermore, findings from this dataset indicate less activation in bilateral occipital cortex during a DT task in highly creative versus less creative individuals (Japardi et al., 2018). Related work by Chrysikou et al. (2020) described patterns of neural activation between eminent and non-eminent thinkers; despite showing no behavioral differences, eminent thinkers engaged more temporoparietal and less occipital areas than did controls during a creative generation task. Additionally, findings from Lotze et al. (2014) show reduced interhemispheric connectivity in highly verbally creative individuals compared with less verbally creative individuals.

A second aim is to link creative experts’ functional connectivity to a uniquely creative behavior other than DT: vividness of imagination. Imagination refers to the ability to mentally construct and manipulate images that are not directly present to the senses. Imagination is closely associated with creativity, drawing upon semantic (Abraham & Bubic, 2015) and episodic memory processes (Devitt et al., 2017). Numerous studies conducted in the context of research on episodic memory and future thinking have described the striking cognitive and neural similarities between remembering past experiences and imagining future or other hypothetical experiences (Schacter et al., 2012, 2017). Emerging behavioral and neuroimaging research point to a conjunction between episodic retrieval, future imagination, and divergent creative thinking by identifying common engagement of the hippocampus and default network regions (Beaty et al., 2018; Madore et al., 2019; Thakral et al., 2020). Additionally, DT has been linked with the ability to vividly imagine novel and specific future events (Addis et al., 2016; Thakral et al., 2021). Behavioral studies have provided evidence for an association between vividness of mental imagery and divergent thinking (Forisha, 1978; Gonzalez et al., 1997). Together, these studies highlight the role of constructive episodic processes in creative thinking.

Meyer et al. (2019) examined whether creative experts might be distinguished in their ability to vividly imagine the future. Across two behavioral studies and one neuroimaging study, the authors compared the vividness of *proximal* (i.e., considering what you might do tomorrow) versus *distal* (i.e., considering what life might be like next century) simulation in creative experts and age-matched controls. In both groups, vividness of proximal simulation involved increased medial prefrontal cortex activity. By contrast, creative experts reported having more vivid distal simulations than did controls, and they showed increased activity of the dorsal medial subnetwork of the DN compared with controls while doing so. The dorsal medial subnetwork includes dorsal medial prefrontal cortex, the temporoparietal junction, along with swaths of inferior frontal and lateral temporal cortex as key structures (Andrews-Hanna et al., 2010) and has been linked to several processes that call on high levels of abstraction, such as semantic processing and mentalizing. Meyer et al. (2019) therefore concluded that an ability to construe abstract thoughts may link creativity and distal simulation. Creative experts in the Meyer et al. (2019) study showed higher connectivity within the dorsal medial DN at rest compared with controls; however, this atlas-based approach is limited, with only the DN tested. This work leaves open the possibility to study whole-brain, voxel-level networks to capture salient features of creative experts’ brains.

Building upon our recent work, we revisit the resting-state fMRI data from Meyer et al. (2019), implementing a high-resolution graph theory approach to more precisely characterize global network features of creative thinkers. As with previous analyses (Orwig et al., 2021), a voxel-level cortical hub strategy was used to identify connectivity differences between creative experts and controls without introducing any regional priors. We hypothesize that creative experts will display brain centrality changes in visual areas relative to controls. As creatives showed task-evoked activity differences specific to distal simulation, we also test whether resting-state connectivity may differentially relate to vividness of distal simulation in creatives versus controls. Linking this finding to higher vividness of distal simulations would serve as evidence that highly creative people may more readily silo visual network connectivity and highlight differential patterns of connectivity between creative experts and controls.

## METHODS

### Sample

The present research uses behavioral and neuroimaging data previously published in Meyer et al. (2019). We analyze resting-state fMRI data from 53 participants (27 creative experts, 26 controls). Creative experts are defined as any individual who either has been recognized by an award for their creative work, held a position at a prestigious institution known for excellence in a creative domain, or attained commercial success in a creative domain. Specifically, writers, actors, and directors were targeted to capture a range of creative expertise, while still ensuring that creative experts had experience imagining distant times, places, and perspectives. Past research showed that writers and actors/directors demonstrated superior distal simulation skills relative to visual artists; furthermore, creative experts significantly outperformed controls on divergent thinking measures, confirming that “real-world” experts are in fact more creative than controls (Meyer et al., 2019). The sample also includes a control group, targeting professionals working in the legal, medical, and financial industries, based on past work that identified individuals in these professions as scoring in the mid-to-low range on standardized creativity assessments (Beketayev & Runco, 2016). Groups did not vary in age (mean creative experts = 36.08 years, *SD* = 9.85 years; mean controls = 33.73 years, *SD* = 7.32 years, *t*(50) = 0.98, *p* = 0.33) or gender (*χ*2(1) = 0.03, *p* = 0.88, *w* = 0.001). Participants provided informed consent in accordance with the Princeton University Institutional Review Board (IRB). All materials and data are available on Open Science Framework (https://osf.io/cy8wt/).

### Distal Simulation Task

Prior to scanning, a subset of participants (*n* = 45; 23 creative experts, 22 controls) completed a distal simulation task, in which they were shown a simulation prompt in the temporal domain (e.g., imagine what the world will be like in 500 years). Participants were shown the prompt for two minutes and instructed to imagine the experience and write a description of their simulation. To derive a subjective measure of vividness, participants then rated the quality of their distal simulations in response to four questions: (i) How vividly did you imagine the experience? (ii) To what extent did you see what you imagined in your mind’s eye? (iii) To what extent did you feel immersed in what you imagined? and (iv) How difficult was it for you to imagine the experience (reverse scored). Participants responded using a 1 (not at all) to 100 (extremely) sliding scale. A composite score of distal simulation vividness was computed as the average response value across these questions.

### MRI Acquisition and Preprocessing

Scanning was conducted at the Princeton Neuroscience Institute on a 3T Siemens Prisma MRI system with a 64-channel head coil. High-resolution T1 scans (MP-RAGE; TR/TE = 2,300/2.27, flip angle = 8°, 256 × 256 matrix, 1 mm thick, 25 0mm FoV) were acquired for anatomical normalization. Resting-state functional scans were acquired with a T2*-weighted echo-planar plus sequence with 69 interleaved slices (TR/TE = 1,500/27ms, flip angle = 75°, 96 × 48 matrix, 2 mm thick, 192 mm FoV; multiband acceleration factor = 3).

MRI data for both anatomical and functional images were preprocessed using FMRIB Software Library v5.0.7 (FSL) and MATLAB 2017a (MathWorks Inc., Natick, MA). The anatomical and functional preprocessing pipelines were adapted from previous work (Diez et al., 2019). The anatomical T1 preprocessing included the following: reorientation to right-posterior-inferior (RPI) with fslreorient2std; alignment to anterior and posterior commissures with a custom script; skull stripping using MNI brain mask template projected to individual space; gray matter, white matter, and cerebrospinal fluid segmentation using FMRIB's Automated Segmentation Tool (FAST) (Zhang et al., 2001); and computation of nonlinear transformation between individual skull-stripped T1 and 2-mm resolution MNI152 template images using the FSL fnirt tool. The functional MRI preprocessing pipeline included the following: slice time correction using Slicetimer; reorientation to RPI with fslreorient2std; realigning functional volumes within runs with rigid body transformations (six-parameter linear transformation); computation of the transformation between individual skull-stripped T1 and mean functional images using boundary-based registration with flirt; intensity normalization; and removal of confounding factors from the data using linear regression, including 12 motion-related covariates (rigid motion parameters and its derivatives), linear and quadratic terms, and five components each from the lateral ventricles and white matter. Global signal regression was not applied owing to the spurious correlations this approach can introduce (Murphy et al., 2009). Transformation of resting-state data to MNI space was performed applying the resulting transformation of concatenating the transformation from functional to structural and from structural to MNI. Spatial smoothing with an isotropic Gaussian kernel of 6-mm FWHM and band-pass filtering (0.01–0.08 Hz) to reduce low-frequency drift and high-frequency noise were also applied. Using in-house MATLAB scripts, head motion was quantified using realignment parameters obtained during image preprocessing, including three translation and three rotation estimates. Scrubbing of time points with excess head motion and interpolation of all time points with Jenkinson framewise displacement greater than 0.2 mm was applied. No participants demonstrated excessive head motion; thus, none were removed from the study based on these criteria. The distributions of the correlations across time series were reviewed for possible contamination. No outliers were observed.

### Weighted Degree Functional Connectivity Analysis

Functional connectivity was computed at the individual level using whole-brain voxel-level weighted degree (WD) analysis. WD is a measure of centrality, computed as the sum of the strengths of functional connections between each voxel and the rest of the brain (Bullmore & Sporns, 2009). This centrality measure captures the global features of the networks, which are thought to be relevant in exceptionally creative people (Anderson et al., 2022). WD is one of many possible measures to capture centrality in functional connectivity. Building upon recent findings of WD connectivity associated with individual differences in creativity (Orwig et al., 2021), we applied the same analysis in this context to detect cortical hubs associated with creative expertise. Pearson correlation coefficients were used to calculate the connectivity matrices of each participant using the time series of all cortical gray matter voxels. An r-to-z Fisher transformation was applied to the resulting correlation matrix, and negative values were removed because of their controversial interpretation (Qian et al., 2018). To minimize noise, we considered only the most significant links using a false discovery rate (FDR) at q-level less than 0.005 (Benjamini & Hochberg, 1995). After obtaining a high-resolution 39,080 × 39,080 connectivity matrix for each participant, we summed all the weighted connections of each voxel to generate the WD adjacency matrix. This adjacency matrix was transformed into a brain map and projected on cortical surfaces showing the extent to which each voxel is functionally connected to the rest of the brain.

### Integration and Segregation

Research into the functional composition of network assembly has revealed a modular organization—networks are composed of densely connected modules, or communities, associated with specific cognitive functions, and cortical hubs that integrate information across communities (Bassett & Bullmore, 2006; Bullmore & Sporns, 2009; Diez et al., 2015). Measures of segregation quantify the presence of communities within the overall brain network, whereas integration captures the brain’s ability to rapidly combine information between communities (Rubinov & Sporns, 2010). To examine group differences in segregation and integration, we organized gray matter voxels with the 17-network solution from Yeo et al. (2011). If the link’s start and end voxels belonged to the same resting-state network, the link was then classified as a segregated link; otherwise, the link was classified as an integrated link. For each participant, we computed the WD of segregated and integrated links separately obtaining two connectivity maps. Higher values in segregated connectivity maps indicate that a given voxel has a higher number of strong functionally connected links to other voxels within the same functional network, whereas higher values in integrated functional connectivity maps indicate that the voxel is an important hub for integrating information between networks.

### Statistical Analysis

At the behavioral level, we conducted univariate linear regression to test for group differences in distal simulation vividness between creative experts and controls. We report the regression parameters (*t* and *p* statistics) for this analysis, with a significance threshold of *α* = 0.05. At the neuroimaging level, general linear models were used to compute the group difference between creative experts and controls for whole-brain WD maps, segregation maps, and integration maps. Additionally, general linear models were used to compute the association between WD and distal simulation vividness within creative experts and control groups. Whole-brain correction for multiple comparisons was computed using Monte Carlo simulation with 10,000 iterations to estimate the probability of false positive clusters with a two-tailed *p* value less than 0.05 (3dClustSim, afni.nimh.nih.gov). First, we generated the residual of the statistics and used it to estimate the spatial autocorrelation present in our data. Then, we computed null models by generating 10,000 random maps with the estimated spatial autocorrelation (3dClustSim). These null models were used to evaluate the likelihood of getting a brain cluster of a particular size for a *p* value less than 0.05 by chance. These data were then used to remove all clusters smaller than the estimated size. Cortical surfaces were visualized using the population-average landmark and surface-based projections of CARET software (Van Essen, 2005). Surface images were displayed using a color scale based on T-scores.

## RESULTS

### Connectivity Patterns Associated With Creative Expertise

**Figure 1.**
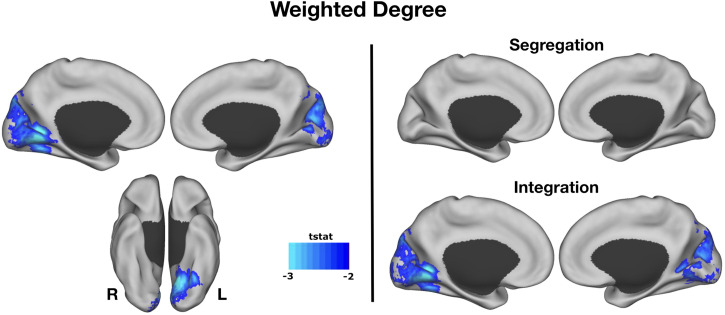
Weighted degree. WD connectivity across the medial visual cortex was lower in creative expertise compared with controls. Segregation maps did not differ between creative experts and controls; however, integration maps reveal a negative association between connectivity of medial visual cortex and creative expertise.

We performed whole-brain WD analysis to identify group-level connectivity differences between creative experts and controls. Creative experts showed lower WD connectivity in the medial visual cortex compared with controls (Figure 1). Next, we examined whether segregation or integration of networks was driving this effect. Segregation maps did not differ between creative experts and controls; however, integration maps reveal a negative association between connectivity of medial visual cortex and creative expertise. In sum, these results indicate that, relative to controls, creative experts have reduced connectivity between visual cortex and the rest of the brain during resting state.

### Connectivity Patterns Associated With Distal Simulation Vividness

**Figure 2.**
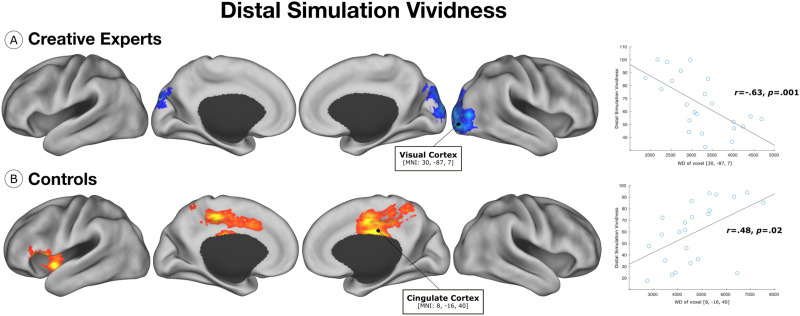
Distal simulation vividness. (A) Within the group of creative experts, WD of voxels in the lateral visual cortex were negatively associated with distal simulation vividness. (B) Within the control group, WD of voxels in the cingulate cortex and left anterior insula were positively associated with distal simulation vividness.

Behavioral analysis revealed no significant differences between creative experts and controls in relation to vividness of distal simulation (*t* = 1.09, *p* = 0.28). Given that Meyer et al. (2019) report significant differences in a larger behavioral sample, it is likely that the present sample lacked sufficient statistical power to detect these behavioral differences. To examine the relationship between functional connectivity and distal simulation vividness, we performed two independent linear regression analyses within groups of creative experts and controls. Results indicate a negative association between WD in the lateral visual cortex and distal simulation vividness in creative experts (Figure 2A). Conversely, we find a positive association between WD of voxels in the cingulate cortex and left anterior insula and distal simulation vividness in controls (Figure 2B). These findings reveal differential patterns of connectivity, between creative experts and controls, associated with vividness of distal simulation.

## DISCUSSION

This study aimed to characterize WD connectivity associated with creativity. Our findings indicate that creative experts show reduced WD connectivity of primary visual areas to the rest of the brain at rest compared with controls. This finding extends past work describing individual differences in continuous creative performance within the general population to a group difference present in a highly expert sample of creatives (Orwig et al., 2021). Moreover, we found that reduced WD connectivity to lateral visual cortex was associated with more vivid distal simulations in creative experts. Taken together, these findings resemble a similar pattern of connectivity associated with creative expertise and distal imagination.

One interpretation of these findings is that in the absence of an external task—namely, during resting state—highly creative people are more prone to engage in internally directed cognition, tuning out sensory information from the present environment. Perceptual decoupling, first described by Schooler et al. (2011), refers to the capacity to disengage attention from perception during mind wandering. Previous studies of creative cognition have found extended deactivation in occipital cortex associated with internal attention (Benedek et al., 2016). The present findings highlight patterns of reduced WD connectivity to visual cortex in highly creative people. It may be the case that creative experts are engaged in more internally directed cognitive processes during resting state and this manifests in reduced connectivity to the primary visual areas, compared with controls. An alternative explanation of these results is that involvement in creative pursuits somehow alters the functional organization of resting-state networks, resulting in the observed reduction in connectivity. It is a well-established phenomenon that repeated exposure to the same stimulus results in diminished response in the human visual cortex. Repetition suppression, the relative attenuation of neural signal evoked by repeated presentation of a stimulus, has been investigated extensively using fMRI (Fritsche et al., 2020; Kourtzi & Kanwisher, 2001; Larsson & Smith, 2012). In the context of the present findings, perhaps the observed negative association between creative expertise and visual cortex connectivity is a consequence of training in a creative domain. According to this view, years of experience with writing, acting, or directing may lead to functional changes in the resting-state network organization. These explanations need not be mutually exclusive: It is possible that creative experts engage in more internally driven thought at rest *and*, in doing so, modify connectivity of visual cortex. Future work aimed at disentangling the two—either by probing thought content at rest or by introducing a novel visual paradigm to examine rates of repetition suppression—will be fruitful in understanding why this functional property is unique to creatives.

Despite the long-standing and intuitive connection between imagination and creative thought, the precise roles of imagery and distal simulation in the creative process remains an open question. While some studies have found a correlation between subjective imagery vividness and creativity (Campos & Pérez, 1989; Shaw & Belmore, 1982), others have failed to observe a reliable association (LeBoutillier & Marks, 2003). Vividness ratings did not differ between the creative experts and controls on which we report here; however, Meyer et al. (2019) did report a difference in their larger behavioral sample. It is likely, then, that the present sample lacked sufficient statistical power. Using this sample, Meyer et al. (2019) reported that creative experts engage regions of the dorsal medial default network during distal simulation more than controls and show higher connectivity within this subnetwork at rest. While Meyer et al. (2019) only tested for group differences within the default network, their results suggested altered brain activity subserving distal simulation across groups that could be detected within functional connectivity patterns at rest. One could argue that failure to observe a behavioral difference in this subsample undermines the logic of comparing functional connectivity between creative experts and controls. It is possible, however, that separable neural resources result in comparable behavior. A prime example of this can be seen in the healthy aging literature, where high-performing older adults recruit additional brain resources to reach comparable performance as young adults on working memory tasks (Cabeza et al., 2002, 2004; Park & Reuter-Lorenz, 2009; Reuter-Lorenz et al., 2000). For this reason, it is essential to look at the correlations within the entire sample as well as separately within each group. Although we acknowledge that the absence of a behavioral difference in our subsample limits to some extent the conclusions that we can draw, by describing associations with distal simulation vividness, within groups of creatives and controls, we provide novel insights into the relationship between imagination and creative expertise.

We therefore examined whether vividness would differentially associate with resting-state functional connectivity across groups. We find that vividness for distal future simulation was negatively associated with connectivity to lateral occipital cortex, but only in creative experts. Numerous studies have reported that visual imagery evokes activation in early visual cortex, though others fail to observe this effect (Kosslyn & Thompson, 2003). Here we find that a distinguishing characteristic of the creative brain, namely lower WD connectivity of visual cortex, may support more vivid distal simulation. This is in contrast to healthy controls, wherein higher vividness ratings were associated with greater WD connectivity between regions of the ventral attention network and the rest of the brain. We focused our analysis exclusively on distal simulation, rather than proximal simulation vividness (which was collected with different procedures, while participants were in the scanner) because past work indicates that imagination of distal future events is directly relevant to creativity (Meyer et al., 2019). Although these findings should be replicated in a larger sample, they suggest diverging brain patterns that may be readily recruited for imagination of the far future. If it is the case that creative people more vividly imagine events in the distant future, it could be speculated that, in order to do so, they must attenuate sensory input from their immediate surroundings.

The present research uses resting-state fMRI data to describe WD connectivity of the brain at rest. It should be noted that there is a considerable amount of noise inherent in working with resting-state data, given that we are not able to control for participant mood or state of mind during scanning. With these limitations in mind, we utilize graph theory metrics in weighted networks, which have been shown to have robust within-subject reproducibility (Ran et al., 2020). Resting-state functional connectivity is thought to reflect the repeated use of circuits during different tasks and has strong correspondence to task-based connectivity (Stevens & Spreng, 2014). It may be the case that differences in connectivity at rest speak to group differences in how creatives and controls engage in simulation tasks, which may in turn be reflected in resting-state architectures. While future studies may seek to replicate these findings in larger samples of creative experts, this data-driven approach offers initial evidence that connectivity differences at rest may reflect brain-related changes associated with creative expertise. Additionally, assessment of more laboratory measures (e.g., trait-level mind wandering, openness to experience) in future samples of creative experts could advance understanding of the behavioral correlates of creativity. Further analysis of the complex interplay between imagination, vividness, and visual cortex promises to offer new insights into the neurocognitive basis of creative thinking.

## AUTHOR CONTRIBUTIONS

William Orwig: Conceptualization; Formal analysis; Investigation; Project administration; Visualization; Writing – original draft; Writing – review & editing. Roni Setton: Methodology; Writing – review & editing. Ibai Diez: Formal analysis; Methodology; Writing – review & editing. Elisenda Bueichekú: Writing – review & editing. Meghan L. Meyer: Data curation. Diana I. Tamir: Data curation; Funding acquisition. Jorge Sepulcre: Funding acquisition; Methodology; Supervision; Writing – review & editing. Daniel L. Schacter: Funding acquisition; Supervision; Writing – review & editing.

## FUNDING INFORMATION

Jorge Sepulcre, National Institutes of Health, Award ID: R01AG061811. Jorge Sepulcre, National Institutes of Health, Award ID: R01AG061445. Daniel L. Schacter, National Institute on Aging (https://dx.doi.org/10.13039/100000049), Award ID: AG008441. Diana I. Tamir, John Templeton Foundation (https://dx.doi.org/10.13039/100000925).

## TECHNICAL TERMS

Divergent thinking: The ability to generate novel ideas to open-ended problems.
Vividness: Degree of detail present in mental imagery.
Distal simulation: Imagination of events in the distant future.
Weighted degree: Graph theory measure that quantifies the relative prominence of each node within the whole network architecture.
Perceptual decoupling: The capacity to disengage attention from perception.
