# Supplementary figures and images for: Creativity at rest: Exploring functional network connectivity of creative experts
Source: Netw Neurosci. 2023 Oct 1;7(3):1022–33. doi: 10.1162/netn_a_00317 (PMC10473280; doi:10.1162/netn_a_00317)

## SUPPLEMENTARY MATERIALS

*Supplementary Figure 1*

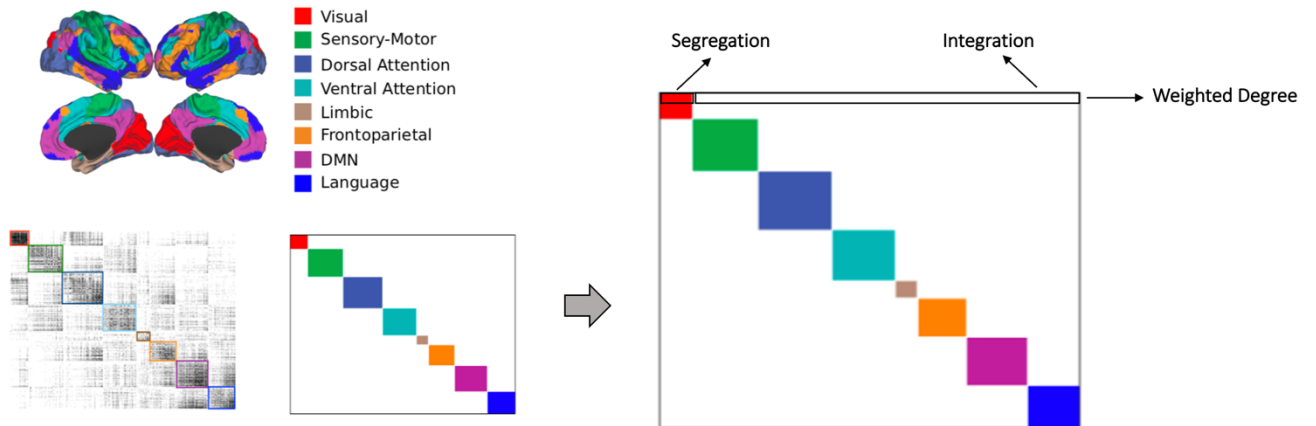

Supplement: Supplementary file 1 [file netn-7-3-1022-s001.pdf]
